# A Deep Learning Strategy for Automatic Sleep Staging Based on Two-Channel EEG Headband Data

**DOI:** 10.3390/s21103316

**Published:** 2021-05-11

**Authors:** Amelia A. Casciola, Sebastiano K. Carlucci, Brianne A. Kent, Amanda M. Punch, Michael A. Muszynski, Daniel Zhou, Alireza Kazemi, Maryam S. Mirian, Jason Valerio, Martin J. McKeown, Haakon B. Nygaard

**Affiliations:** 1 Department of Electrical and Computer Engineering Capstone, University of British Columbia, Vancouver, BC V6T 1Z4, Canada; amelia.casciola@gmail.com (A.A.C.); sebastiano.carlucci@gmail.com (S.K.C.); amandampunch@gmail.com (A.M.P.); michael.ampm@gmail.com (M.A.M.); danielzhou4970@gmail.com (D.Z.); 2 Djavad Mowafaghian Centre for Brain Health, Division of Neurology, University of British Columbia, Vancouver, BC V6T 1Z3, Canada; bkent@sfu.ca (B.A.K.); maryam.mirian@ubc.ca (M.S.M.); jvale093@gmail.com (J.V.); 3 Department of Psychology, Simon Fraser University, Burnaby, BC V5A 1S6, Canada; 4 Center for Mind and Brain, Department of Psychology, University of California, Davis, CA 95618, USA; kazemi@ucdavis.edu

**Keywords:** deep learning, EEG headband, sleep staging, machine learning, neurodegenerative disease, sleep

## Abstract

Sleep disturbances are common in Alzheimer’s disease and other neurodegenerative disorders, and together represent a potential therapeutic target for disease modification. A major barrier for studying sleep in patients with dementia is the requirement for overnight polysomnography (PSG) to achieve formal sleep staging. This is not only costly, but also spending a night in a hospital setting is not always advisable in this patient group. As an alternative to PSG, portable electroencephalography (EEG) headbands (HB) have been developed, which reduce cost, increase patient comfort, and allow sleep recordings in a person’s home environment. However, naïve applications of current automated sleep staging systems tend to perform inadequately with HB data, due to their relatively lower quality. Here we present a deep learning (DL) model for automated sleep staging of HB EEG data to overcome these critical limitations. The solution includes a simple band-pass filtering, a data augmentation step, and a model using convolutional (CNN) and long short-term memory (LSTM) layers. With this model, we have achieved 74% (±10%) validation accuracy on low-quality two-channel EEG headband data and 77% (±10%) on gold-standard PSG. Our results suggest that DL approaches achieve robust sleep staging of both portable and in-hospital EEG recordings, and may allow for more widespread use of ambulatory sleep assessments across clinical conditions, including neurodegenerative disorders.

## 1. Introduction

It is increasingly recognized that sleep abnormalities often accompany neurodegenerative disorders and in some cases are considered a core manifestation of the disease [1]. Research is now focused on determining whether sleep disturbances antedate disease onset and/or represent a biomarker of disease progression [1].

The gold standard in sleep assessment is polysomnography (PSG). However, there are several factors that limit the usefulness of PSG for studying sleep in patients with neurodegenerative diseases. First, it is relatively expensive, and thus many PSG studies are statistically under-powered. Second, the unnatural environment and discomfort associated with the numerous electrodes and wires may disturb the subject, and results using PSG may therefore not accurately reflect sleep in the home environment [2]. Third, patients with dementia are prone to delirium and it is not always ethically feasible for all clinical populations to undergo inpatient PSG [3]. Finally, the proportion of subjects in need of formal sleep assessments outweighs the capacity of accredited PSG sleep laboratories, limiting access to diagnostic services.

Because of these limitations there is a desire to move toward inexpensive, comfortable, in-home sleep measurements. However, with portable EEG measurement devices, the data quality is often a key concern. PSG provides high-quality data collected under tightly controlled conditions from multiple simultaneous recordings, monitored in real time by trained technicians. In contrast, portable headsets are used in uncontrolled environments and are limited in the type and amount of data collected (e.g., number of simultaneous EEG electrodes and physiological measurements). A common form of in-home EEG measurement device is a headband (HB), where the electrodes are placed on a fabric band wrapping the circumference of the subject’s head. In headbands, electrodes are often placed on a subject’s face and forehead, where hair is not a concern. This concentration over frontal regions causes the data to be susceptible to corruption by ocular artifacts created by eye blinks, flutters, and eye movements as well as muscle artifacts from the frontalis muscle [4]. However, this electrode placement functions remarkably well in detecting slow wave power [5], which is critically important in the assessment of Alzheimer’s disease [6].

Computer-aided diagnosis has been used effectively for some time in various healthcare applications [7,8,9,10,11], and several deep learning (DL) methods and neural network architectures have been proposed to automate sleep stage scoring using single, double, or multichannel PSG EEG data [12]. During the sleep staging process, the EEG signal is divided into periods of Wake, rapid eye movement (REM) and non-REM sleep (NREM). The NREM stage is further split into stages N1, N2 and N3. Convolutional neural networks (CNN), recurrent neural networks (RNN) or a mixture of both have been applied with a high degree of success in EEG processing for sleep stage classification [13,14,15]. Convolutional neural networks are particularly efficient at extracting time-invariant and localized information [16]. In EEG processing, convolutional layers replace the task of manual feature engineering by relying on the network’s training ability to learn relevant features. Whereas recurrent neural networks impose a structure to learn relationships between consecutive data points in a sequence.

However, current machine learning (ML) models built for PSG data typically fail to generalize well on EEG data collected from a HB. The significant difference in the distribution between HB and PSG data is reinforced by using a covariate shift similarity test [17]. The test identifies a covariate shift between two datasets based on whether the origin of any sample from either set can be correctly identified. A simple classifier was trained to predict whether a sample was collected with PSG or HB and was able to correctly predict the origin 99% of the time. This highlights the differences in characteristics and distribution between both types of EEG. These differences explain why models built for PSG cannot necessarily be applied to HB data with an expectation of the same degree of success. Here we present a model that is more robust than current automated sleep staging solutions and is able to perform sleep staging accurately on both PSG and HB data.

Limited studies applying ML approaches to HB recordings have been promising. We found only one publicly available HB dataset, collected using the Dreem Headband (Dreem, San Francisco, CA, USA) [18], and only a handful of studies on sleep staging with HB EEG [2,5,19,20,21,22]. For example, Levendowski and colleagues [22] compared simultaneous recordings from 47 participants, using a commercially available three-channel EEG recording device (Sleep Profiler, Advanced Brain Monitoring, Carlsbad, CA, USA) and PSG, and found that the overall agreement between the technologist-scored PSG and the automated sleep staging of the EEG device was 71.3% (kappa = 0.67). Given that the five technologists in the study had an overall interscorer agreement of 75.9% (kappa = 0.70), it was concluded that the autoscored frontal EEG was as accurate as human-staged PSG, except for sleep stage N1. Similarly, Lucey and colleagues [5] compared the same three-channel EEG device (Sleep Profiler, Advanced Brain Monitoring, Carlsbad, CA, USA) with simultaneous PSG in 29 participants and found similar agreement (kappa = 0.67). However, in this study, the two technologists had extremely high interscorer agreement for the PSG data (kappa = 0.97, 80.8–98.8% depending on stage), so there was a substantial drop compared to the autoscored EEG. Of the HB sleep staging solutions studied, only one attempted deep learning. Arnal and colleagues achieved an accuracy of 83.5%, compared to the interscorer agreement of 86.4% [2], using a long short-term memory (LSTM)-based model. This model used 25 nights of HB EEG collected with the Dreem Headband, and incorporated heart rate, respiration rate and respiration rate variability into their algorithm.

Here, we present an automatic sleep staging method for two-channel HB EEG data based on a CNN + LSTM architecture. The model was trained and tested with data from 12 overnight EEG recordings from 12 different participants. We achieve a mean accuracy of 74% when sleep staging HB data, compared to 68% when using a traditional ML pipeline. Additionally, when balanced accuracy is taken into consideration, the deep learning method outperforms traditional automated techniques by 20%. The increase in balanced accuracy implies consistent performance across all sleep stages.

Our model’s predictive ability on low-quality data indicates its potential in clinical and research applications. By advancing the usability of EEG headband data, this model can dramatically expand formal sleep staging in an ambulatory setting, paving the way for larger studies in key populations such as Alzheimer’s disease. Additionally, the DL model’s predictive power may also benefit future work on computer-aided diagnosis of neurodegenerative diseases.

## 2. Materials and Methods

### 2.1. Data Collection

The data was collected from a group of 12 subjects overnight at the University of British Columbia Hospital Blackmore Centre for Sleep Disorders. The subjects (6 male, 6 female) were between the ages of 21 and 61. Participants were simultaneously monitored with a PSG setup, as well as a Cognionics 2-channel EEG headband (Cognionics, San Diego, CA, USA). Data collection was approved by Vancouver Coastal Health Authority-UBC and University of British Columbia Office of Research Ethics (ethics protocol H16-00925).

**Figure 1:**
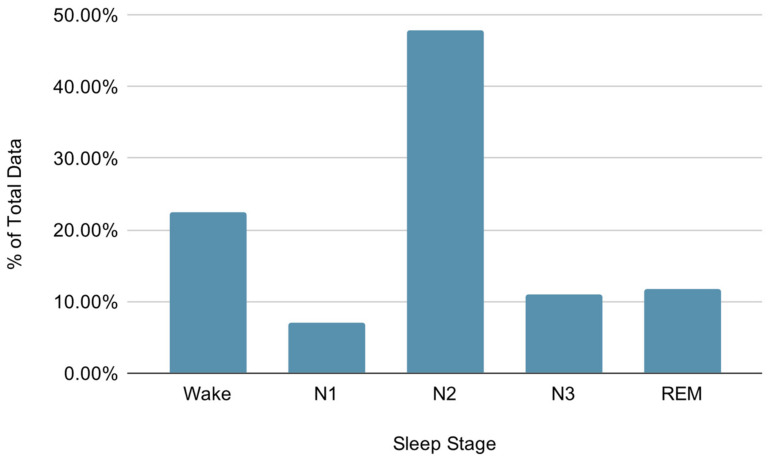
Distribution of sleep stages assigned by the Senior Polysomnographic Technologist (Wake: 22.45%, N1: 6.95%, N2: 47.86%, N3: 11.07%, rapid eye movement (REM): 11.66%).

An overnight attended level 1 PSG was performed using standard electrode montages. This included EEG (channels F3-M2, F4-M1, C3-M2, C4-M1, O1-M2 and O2-M1), electrooculography (EOG), electromyography (EMG), electrocardiography (ECG), oronasal thermal airflow sensor, pulse oximetry, respiratory inductance plethysmography acoustic sensor and audio-equipped video PSG as recommended by American Academy of Sleep Medicine (AASM) criteria (version 2.4, 2017) [23]. The headband setup was performed according to the Cognionics manufacturer instructions [24]. Both PSG and HB EEG recordings used adhesive foam electrodes (Kendall Medi-Trace Mini, Davis Medical Electronics, Vista, USA). The overnight PSG was scored by a Senior Polysomnographic Technologist and interpreted by a board-certified sleep physician using standard AASM criteria based on 6 EEG channels, 2 EOG channels and 1 EMG channel [23]. The manually scored PSG labels were used for both the PSG and HB recordings. To control for any possible temporal misalignment between the two systems, we performed both automatic correlation checking of lagged signals and manual inspection. In Figure 1, we report the distribution of the assigned sleep stage labels. The dataset is highly unbalanced toward the N2 and Wake classes, the N2 class being twice more frequent than the Wake class and roughly seven times more frequent than the N1 class. This label imbalance is to be expected, since the majority of sleep is usually spent in the N2 stage [25].

The HB EEG recordings were taken between nodes F3-A1 (channel 1) and F4-A2 (channel 2). The characteristics of the raw recorded HB data are reported in Table 1.

### 2.2. Data Preprocessing

Slightly different preprocessing pipelines were applied to PSG and HB data, as presented in Section 2.2.1 and Section 2.2.2.

#### 2.2.1. Preprocessing PSG Data

Following the sleep scoring, only two PSG channels (F3-M2 and F4-M1) were used for the remainder of the study, to most closely match the electrode placement of the HB. A Finite Impulse Response (FIR) bandpass filter with cutoff frequencies of 0.5 and 12 Hz was applied to these collected PSG channels. The data were then downsampled to 25 Hz and separated into nonoverlapping 30-s epochs.

#### 2.2.2. Preprocessing HB Data

The HB EEG data was visually inspected to identify segments which were corrupted beyond interpretation due to hardware error and a section of 50 min was removed from one subject. The erroneous epochs were likely due to electrodes losing contact with the skin surface.

The same bandpass filtering and epoching as described in Section 2.2.1 was applied to the HB data.

### 2.3. HB Data Cleaning

**Figure 2:**
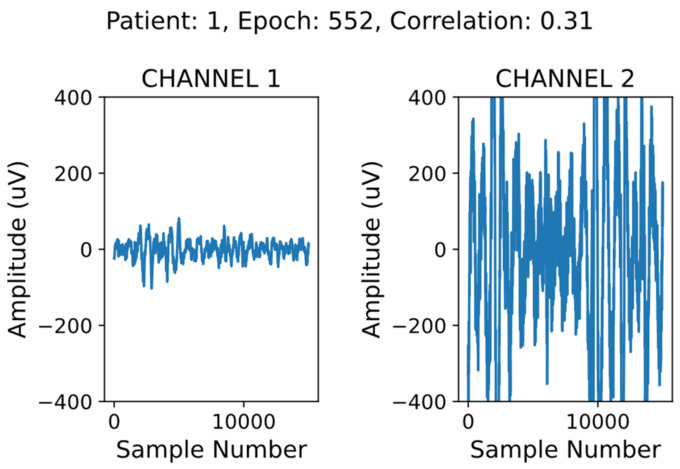
Valid electroencephalogram (EEG) signal shown on the left, corrupted data on the right.

In addition to preprocessing, we implemented a simple data screening algorithm to automatically remove corrupted epochs from the HB data only. An example of a corrupted epoch is shown in Figure 2. There are a few key indicators that the signal on the right (channel 2) is not a valid EEG signal. The first is that the amplitude of the channel 2 signal largely exceeds the amplitude of an average EEG signal (generally below 100 µV) [26]. The second is that there is no visual similarity between the channel 1 and channel 2 signals.

To remove the corrupted data, we filtered the epochs where the correlation between the signals from channel 1 and channel 2 was low (below 0.9), and the mean amplitude of at least one channel was high (above 40 µV). We removed only the data in the corrupted channel, duplicating the valid channel in its place. This method aims to minimize the amount of valid data being discarded, while making use of the expected correlation between mirroring channels [27].

A grid search was performed in order to determine the best correlation and amplitude thresholds. First, ranges of valid amplitude and correlation thresholds were selected by manual parameter adjustment and inspection of the filtered epochs. Next, a grid search was performed on each combination of minimum correlation from 0.6 to 0.9 in steps of 0.1 and maximum channel amplitude from 30 µV to 60 µV in steps of 10 µV. For each of these 16 threshold combinations, we performed leave-one-out cross validation 12 times, once for each subject.

### 2.4. Data Augmentation

**Figure 3:**
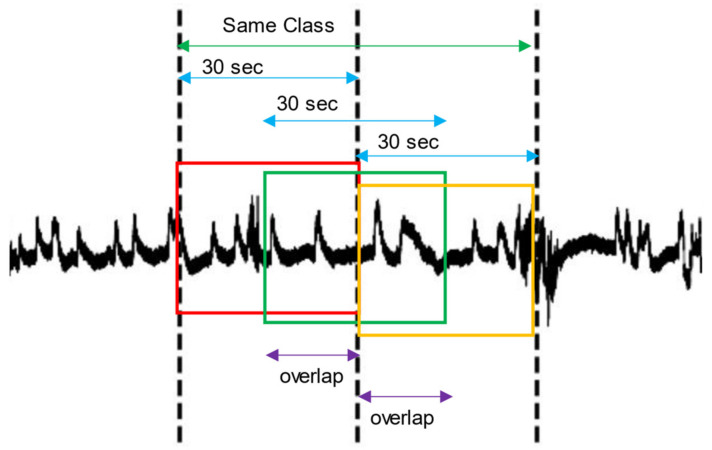
Overlapping windows applied over the EEG signal (black line). The dotted vertical lines delimit two 30-s epochs of the same class. The red, green and yellow rectangles correspond to the newly generated epochs after applying a specified overlap (purple).

It has been shown that data augmentation can substantially increase accuracy when using deep learning for EEG analysis [28]. We implemented overlapping windows in order to artificially increase the size of our dataset, an approach which has yielded promising results in other literature [29,30,31]. In this method, we concatenated all contiguous 30-s epochs of the same sleep stage in the time domain. The concatenated blocks were then redivided into new 30-s epochs, all overlapping by 75%. This process is illustrated in Figure 3. We applied this method only on training data to avoid corrupting the prediction results.

To determine the optimal overlap percentage, we generated artificially augmented datasets using overlap percentages of 75%, 50% and 20%. The optimal overlap percentage was determined after averaging the results of leave-one-out cross validation over the 12 participants.

### 2.5. Deep Learning Model Architecture

**Figure 4:**
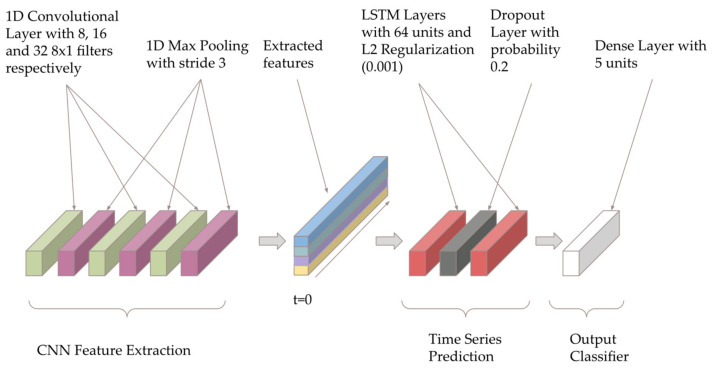
Convolutional and long short-term memory (CNN + LSTM) model architecture inspired by [37].

Based on a review of existing deep learning models for sleep staging using EEG signals, the most promising architectures for low-quality EEG data were CNN and CNN + LSTM models [32,33,34,35,36]. We implemented a CNN + LSTM model based on Bresch’s model for single-channel EEG [37], with the architecture shown below in Figure 4. The model was created using Python 3 and the Keras framework with TensorFlow backend version 2.2, and summarized in Table A1.

The model contains three convolutional layers (to learn the useful spatial information), followed by two LSTM layers (to extract influential temporal information) and a dense layer. The first convolutional layer has eight filters, and this number doubles with each convolutional layer. The two LSTM layers have 64 units, with the final dense layer only containing five, as well as a softmax output activation. The convolutional layers are particularly effective for extracting time-invariant and localized information. Their function in the model is similar to the task of manual feature extraction in traditional machine learning methods. The LSTM layers then impose a structure to learn relationships between the consecutive data points extracted by the CNN layers.

Implementing regularization is a common method of reducing overfitting in deep learning models [38,39]. In our model, the LSTM layers are regularized to by adding a dropout of 0.2. By randomly assigning different nodes to a weight of zero at each iteration, dropout can simulate a range of architectures, as opposed to one static model layout. This minimizes the risk of certain nodes developing large weights based on the training data, reducing generalizability [40]. To further reduce the model’s reliance on individual features we implemented L2 weight decay with parameter 0.001.

### 2.6. Model Training

To guide the training of the model, we use the categorical cross entropy loss function. When the dataset is unbalanced (the distribution is skewed toward some labels), the model’s predictions tend to become biased toward the most common labels. Our dataset is highly unbalanced with the most frequent class, sleep stage 2, comprising 47% of the data. To address this, we apply a per-class weight to the categorical cross entropy loss function:

(1) $$L\left(y,\hat{y}\right)\;=\;-\overset{m}{\underset{i=1}{\sum }}w\left(y_{i}\right)y_{i}\log\left({\hat{y}}_{i}\right)$$

where y and $\hat{y}$ are the true labels and the predictions, respectively, and $L\left(y,\hat{y}\right)$ is the loss. The relative weight of label $y_{i}$ in the dataset (the number of $y_{i}$ labels divided by the number of examples in the dataset) is defined as $w\left(y_{i}\right)$. The parameters of the model are updated at each training iteration using an Adam optimizer [41] with an initial learning rate of 5 × 10^−5^, $\beta_{1}$ = 0.99 and $\beta_{2}$ = 0.999.

Training was performed using leave-one-out cross validation, for 200 epochs per fold, using a batch size of 64. This process was completed using a NVIDIA Titan RTX GPU (Nvidia, Santa Clara, CA, USA), and a 4.0 GHz Intel processor (Intel, Mountain View, USA). Each training epoch took about 3 s to complete, with the entire process taking approximately 2 h.

### 2.7. Traditional Sleep Staging Techniques

As well as using our deep learning model for sleep staging, we also attempted multiple traditional processing pipelines. The highest accuracy of these supervised modeling techniques was achieved using extracted features and an ensemble bagged trees model.

After a similar preprocessing pipeline to that described in Section 2.2, we extracted features in the following categories:

- Frequency domain;
- Time domain;
- Higher-order statistical analysis (HOSA)-based;
- Wavelet-based.

In total, 62 features per EEG channel were extracted and are listed in Table 2. The computed values for the features were normalized using Min-Max feature scaling for a final value between 0 and 1.

As a classic baseline approach, the ensemble-bagged trees model was trained on these features using leave-one-out cross validation.

## 3. Results

### 3.1. Deep Learning Model Performance

**Figure 5:**
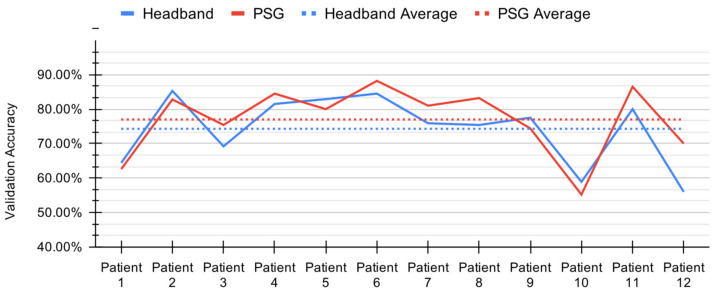
Per-patient validation accuracy during leave-one-out cross validation for the convolutional and long short-term memory (CNN+LSTM) model with headband (HB) and polysomnography (PSG).

Combining the model architecture, data cleaning and data augmentation methods described previously, we performed leave-one-out cross validation for each participant. We achieved a mean prediction accuracy of 74.01% (standard deviation 10.32%) with HB data and 76.98% (standard deviation 10.05%) with PSG data. These results are summarized in Figure 5.

The similarity in sleep staging accuracy achieved with HB and PSG demonstrates this model’s robustness to variance in data source and thus quality. In Table A2, we include the confusion matrices for each subject for both HB and PSG.

Furthermore, we report the average stage-wise performance of the deep learning CNN+LSTM model over 12 folds, also known as the percent correct recall. The results shown in Table 3 highlight the model’s similar performance across sleep stages N2, N3, REM and Wake despite the unbalanced label frequencies. Especially notable is the high accuracy achieved for sleep stage N3, which comprises only 11% of the dataset, and may be particularly important for risks of dementia [6]. The accuracies achieved for stage N1 are considerably lower, as is commonly the case with both manual sleep staging and automated methods [42,43].

To provide an interpretation of the model’s predictive behavior, we followed a similar approach to Chambon and colleagues [13]. We analyzed whether the model learned to discriminate between sleep stages based on frequency bands, which is a common approach among human sleep scorers. For example, the relative spectral density of the δ band (0.5–4 Hz) is often used by human scorers to classify epochs as “deep sleep” (sleep stage N3). We studied how the model’s predictive behavior changes with respect to the following frequency bands: δ (0.5–4 Hz), θ (6–8 Hz) and α + β (>8 Hz). We filtered the test data into the aforementioned frequency bands and passed these individually into the pre-trained model.

**Figure 6:**
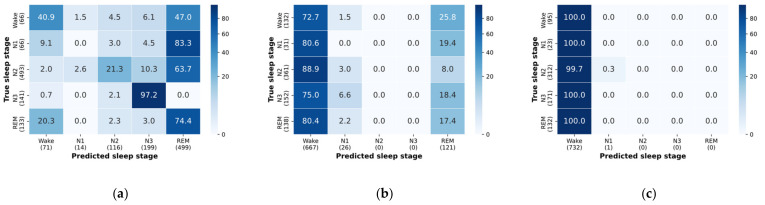
Confusion matrices for inputs passed through the following bandpass filters: (**a**) delta; (**b**) theta; (**c**) alpha and beta.

The model associates higher frequencies with the Wake stage, and its predictive power for sleep stage 3 does not decrease when given the delta band of a signal (Figure 6). These results point to the use of frequency bands for the sleep stage prediction of Wake and N3. However, the results indicate that the model does not learn to classify sleep stages 1 and 2 based on the presence of theta frequencies.

### 3.2. Baseline Model Performance

**Figure 7:**
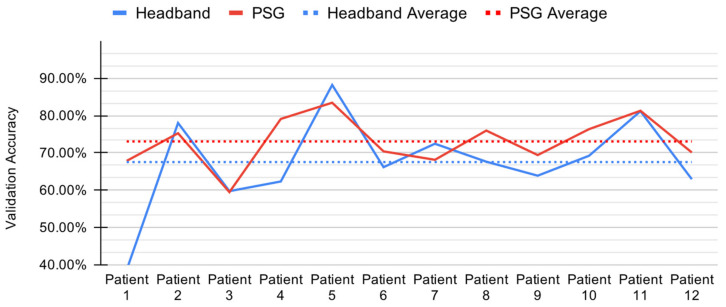
Per-patient validation accuracy during leave-one-out cross validation for the ensemble bagged trees model with headband (HB) and polysomnography (PSG).

The ensemble bagged trees method presented in Section 2.7 achieved an accuracy of 67.53% (standard deviation 11.94%) with HB data, and accuracy of 73.07% (standard deviation 6.46%) with PSG data, as shown in Figure 7.

We report the average stage-wise performance of the ensemble bagged trees method over all 12 folds, shown in Table 4. This model performs best on N2 and Wake stages for both HB and PSG data, while performing comparatively poorly on N1, N3 and REM sleep stages.

The balanced accuracy, which is computed as the mean of all percent recalls of the labels, is also reported with 48.88% and 55.21% on HB and PSG data, respectively.

The low accuracies for N1, N3 and REM and high accuracies for N2 and Wake are justified by the dataset bias toward the N2 and Wake labels. This implies that the ML model is overfitting the dataset, and does not provide any meaningful predictions on the data.

## 4. Discussion

We have shown that a DL sleep staging model achieves 74% accuracy on low-quality headband EEG data, compared to 77% with gold-standard PSG. The model performs well across all sleep stages, leading to a balanced accuracy of almost 20% more than any machine learning sleep staging method attempted. We also show that this model achieves an especially high accuracy for sleep stage N3, with acceptable performance for REM sleep classification, both of which may be highly relevant to the pathophysiology of neurodegenerative disorders [1,6]. These results were attained without any extensive preprocessing or special artifact removal procedures. Our findings address the major barrier preventing more widespread use of HB for ambulatory sleep assessment, largely related to poor performance in automated classification tasks.

### 4.1. Deep Learning Model Comparison

As previously noted, the architecture of our deep learning solution was adapted from a model created by Bresch and colleagues [37]. However, our implementation has a few key differences. Firstly, we reduce overfitting by regularizing the LSTM layers with dropout and weight decay. Secondly, the CNN output in Bresch’s architecture is flattened, which discards the temporal structure of the signal. Instead of flattening the CNN output, we directly pass the time sequence of the extracted features to the downstream LSTM layers.

Another key difference between our model and Bresch’s is the input data. For our use-case, we have two-channel EEG data, downsampled to 25 Hz as opposed to one 100 Hz channel. Finally, due to our substantially smaller dataset, we include a comprehensive data cleaning and data augmentation preprocessing pipeline, before passing the signal to the deep learning model, something which is not done by Bresch and colleagues.

Using Bresch’s architecture before our adjustments resulted in a mean accuracy over 12 folds of 70.83% (standard deviation 10.55%), while after our adjustments, the results improved by almost 4%.

### 4.2. Baseline Model Comparison

We present a comparison between the results of the CNN+LSTM model and the ML model described in the previous sections. The accuracies and balanced accuracies shown in Table 3 and Table 4 demonstrate the deep learning model’s superior adaptability to changes in data quality. The DL model shows a 2% difference between balanced accuracies when trained on PSG versus trained on HB. The ML model, on the other hand, shows a difference of 6%. The smaller gap between PSG and HB results points to a more robust model, indicating that data quality is not a significant variable with respect to sleep staging performance.

When comparing the two models’ results directly, the DL model has a higher accuracy and balanced accuracy both with HB and PSG data. Specifically, we see an additional increase of 6.50% in accuracy and 19.77% in balanced accuracy using HB data, when compared to the ML model. Similarly, we see an additional increase of 3.94% in accuracy and 15.63% in balanced accuracy using PSG data. The higher accuracy indicates an improvement in overall predictive capability, while the higher balanced accuracy indicates an improvement in the model’s generalizability across all sleep stages.

Stage-wise comparison of the model performances show that the DL model’s N2 accuracy decreased slightly from 82.32% on HB (and 88.36% on PSG) to 74.87% on HB (and 77.82% on PSG), in exchange for a significantly greater increase in the under-represented labels (N1, N3 and REM). The largest of these reported increases is in stage N3, where an additional 34.37% on HB data (and 49.37% on PSG data) is gained.

### 4.3. Electrode Comparison

The EEG recordings in this study used gel electrodes that balance ease of use, patient comfort, and signal quality (Kendall Medi-Trace Mini, Davis Medical Electronics, Vista, USA). New developments of dry and semi-dry electrodes have been promising [44,45,46,47], and while we anticipate similar sleep staging results using our DL model with dry and semi-dry electrode variants, additional studies are needed to test this hypothesis.

## 5. Conclusions

We demonstrate that a simplified two channel HB EEG device can be used to accurately stage sleep when combined with a DL data analysis model. The DL model outperforms traditional machine learning approaches, and may allow for wider application of ambulatory sleep assessments across clinical populations, including neurodegenerative disorders.

## Figures and Tables

**Table 1:** Headband data characteristics.

Bandwidth	Sample Rate	Amplifier Gain	Resolution	Noise
0–131 Hz	500 samples/sec	6	24 bits/sample	0.7 μV

**Table 2:** Type and number of features extracted from each EEG channel.

Feature Category	Feature Group	Feature Size
Frequency Domain	RSP	11
	HP	15
	SWI	3
Time Domain	Hjorth	3
	Skewness	1
	Kurtosis	1
HOSA	Bi-Spectrum	20
Wavelet	Relative Power	8
Total Features		62

**Table 3:** Average stage-wise performance over all folds for the deep learning model.

Data	N1	N2	N3	REM	Wake	Accuracy	Balanced Accuracy
**HB**	29.80%	74.87%	84.02%	73.96%	80.60%	74.01%	68.65%
**PSG**	31.08%	77.82%	85.27%	75.38%	84.64%	77.00%	70.84%

**Table 4:** Average stage-wise performance over all folds for the ensemble bagged trees method.

Data	N1	N2	N3	REM	Wake	Accuracy	Balanced Accuracy
**HB**	4.11%	82.32%	49.65%	28.26%	80.03%	67.53%	48.88%
**PSG**	13.13%	88.36%	35.90%	55.17%	83.49%	73.06%	55.21%

## Data Availability

The data presented in this study are available upon request from the corresponding authors. The source code created in this study can be found at: https://bitbucket.org/cap131/headbandsleepscorer/src/master/ (accessed on 2 May 2021).

## Appendix A

**Table A1:** Convolutional and long short-term memory (CNN + LSTM) model summary.

Layer Type	Output Shape	Param #
Conv1D	(None, 2993, 8)	136
Activation (ReLU)	(None, 2993, 8)	0
MaxPooling1D	(None, 997, 8)	0
Conv1D	(None, 990, 16)	1040
Activation (ReLU)	(None, 990, 16)	0
MaxPooling1D	(None, 330, 16)	0
Conv1D	(None, 323, 32)	4128
Activation (ReLU)	(None, 323, 32)	0
MaxPooling1D	(None, 107, 32)	0
LSTM	(None, 107, 64)	24,832
LSTM	(None, 64)	33,024
Dense	(None, 5)	325
Total Params	63,485	
Trainable Params	63,485	
Non-Trainable Params	0	

**Table A2:** Confusion matrices for deep learning model predictions on each subject.

	Headband (HB)	Polysomnography (PSG)
Subject 1		
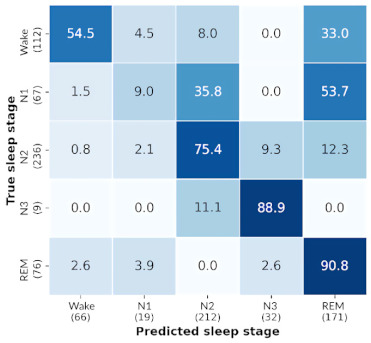
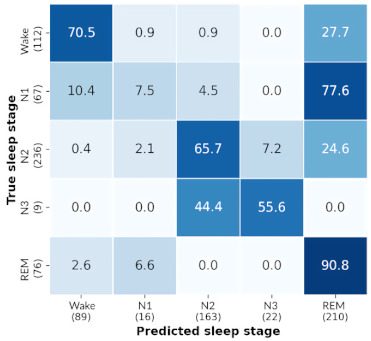
Subject 2		
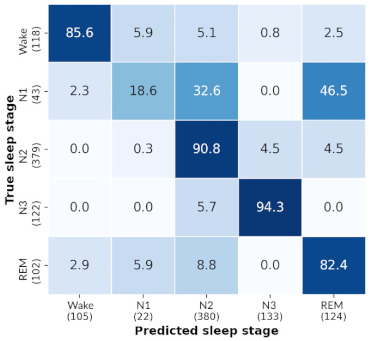
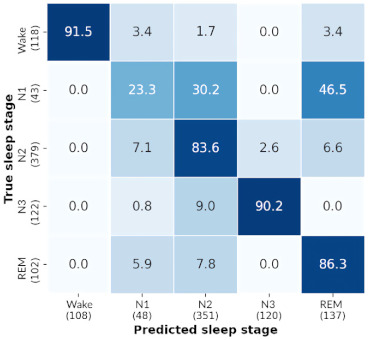
Subject 3		
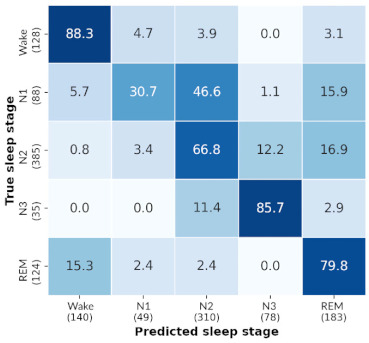
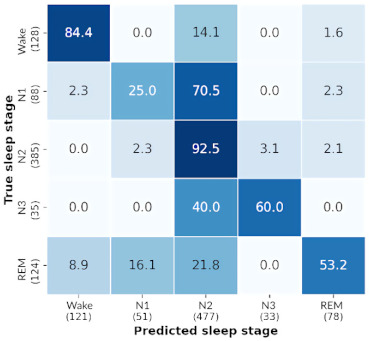
Subject 4		
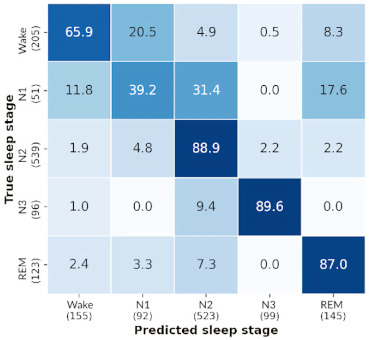
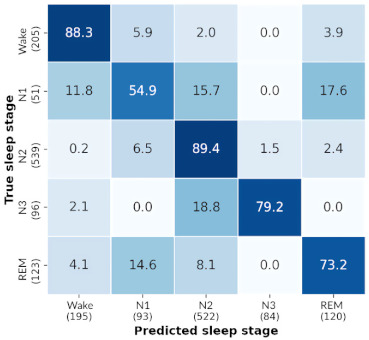
Subject 5		
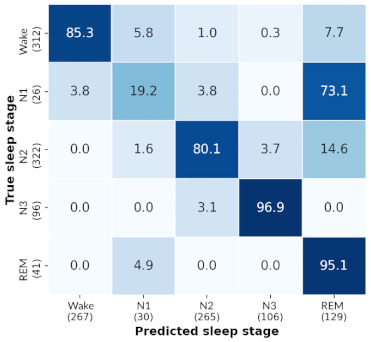
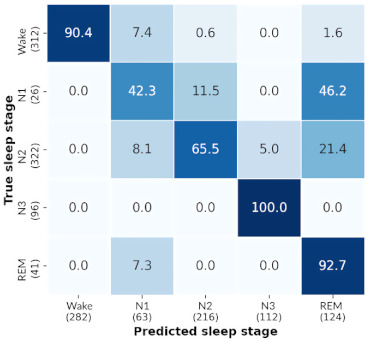
Subject 6		
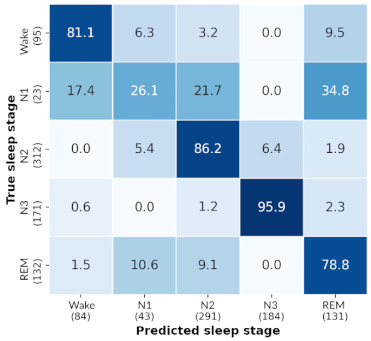
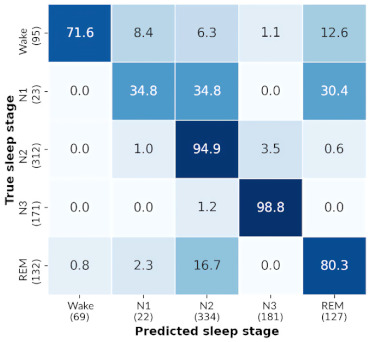
Subject 7		
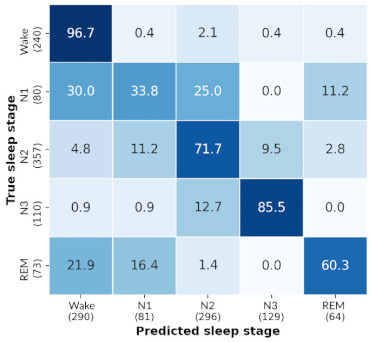
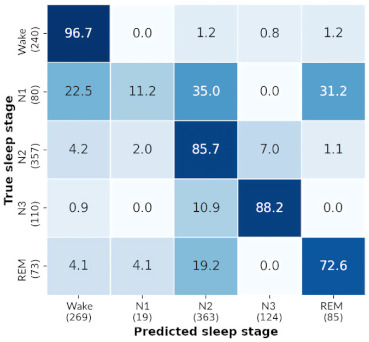
Subject 8		
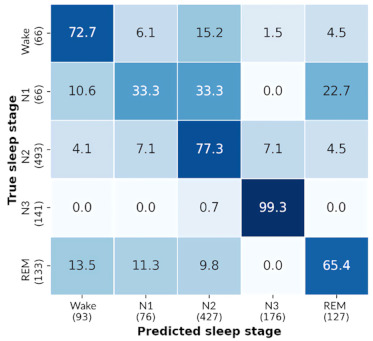
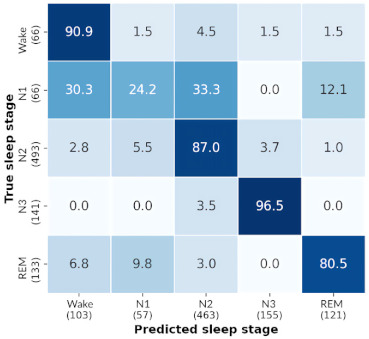
Subject 9		
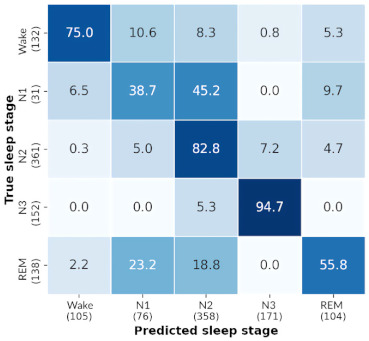
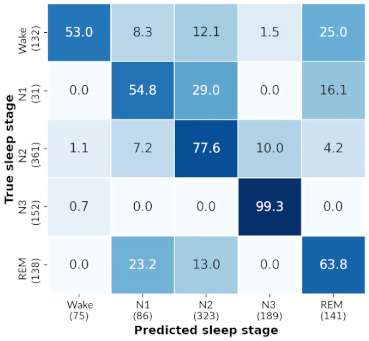
Subject 10		
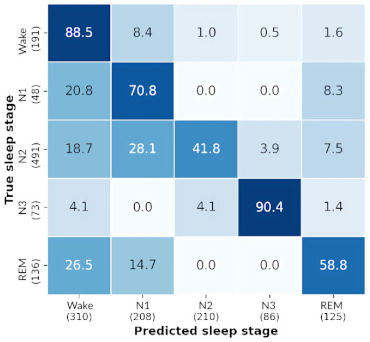
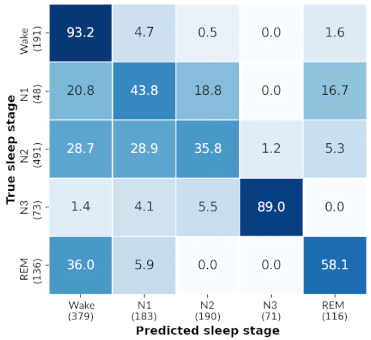
Subject 11		
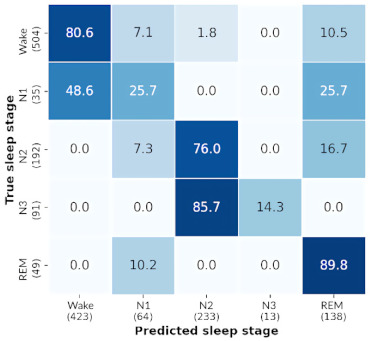
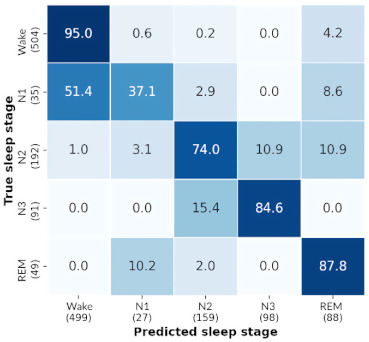
Subject 12		
